# Single-cell analysis of p53 transitional dynamics unravels stimulus- and cell type-dependent signaling output motifs

**DOI:** 10.1186/s12915-022-01290-7

**Published:** 2022-04-11

**Authors:** Jun Xie, Lichun Zhang, Bodong Liu, Xiao Liang, Jue Shi

**Affiliations:** Center for Quantitative Systems Biology, Department of Physics and Department of Biology, Hong Kong Baptist University, 224 Waterloo Road, Kowloon Tong, Kowloon, Hong Kong, China

**Keywords:** p53 network motif, Single-cell dynamics, Cancer variability, Drug sensitivity

## Abstract

**Background:**

To understand functional changes of complex biological networks, mathematical modeling of network topologies provides a quantitative measure of the way biological systems adapt to external stimuli. However, systemic network topology-based analysis often generates conflicting evidence depending on specific experimental conditions, leading to a limited mechanistic understanding of signaling networks and their differential dynamic outputs, an example of which is the regulation of p53 pathway responses to different stress stimuli and in variable mammalian cell types. Here, we employ a network motif approach to dissect key regulatory units of the p53 pathway and elucidate how network activities at the motif level generate context-specific dynamic responses.

**Results:**

By combining single-cell imaging and mathematical modeling of dose-dependent p53 dynamics induced by three chemotherapeutics of distinct mechanism-of-actions, including Etoposide, Nutlin-3a and 5-fluorouracil, and in five cancer cell types, we uncovered novel and highly variable p53 dynamic responses, in particular p53 transitional dynamics induced at intermediate drug concentrations, and identified the functional roles of distinct positive and negative feedback motifs of the p53 pathway in modulating the central p53-Mdm2 negative feedback to generate stimulus- and cell type-specific signaling responses. The mechanistic understanding of p53 network dynamics also revealed previously unknown mediators of anticancer drug actions and phenotypic variations in cancer cells that impact drug sensitivity.

**Conclusions:**

Our results demonstrate that transitional dynamics of signaling proteins such as p53, activated at intermediate stimulus levels, vary the most between the dynamic outputs of different generic network motifs and can be employed as novel quantitative readouts to uncover and elucidate the key building blocks of large signaling networks. Our findings also provide new insight on drug mediators and phenotypic heterogeneity that underlie differential drug responses.

**Supplementary Information:**

The online version contains supplementary material available at 10.1186/s12915-022-01290-7.

## Background

Despite the extensive molecular knowledge that we have for large signaling networks, we still lack effective methods to quantitatively understand and predict their context-specific dynamic outputs. A case in point is the well-known p53 regulatory network, which consists of the key tumor suppressor and transcription factor p53, its upstream regulators, and downstream target genes, and plays a central role in eliciting proper cellular responses to chromosomal damage and various stresses in mammalian cells [1, 2]. Referred to as the “guardian of the genome,” p53 and the extended p53 pathway have been widely studied, revealing multi-level post-transcriptional control over p53 activity via a complex network of signaling kinases, cofactors, and inhibitory partners [3–5]. The plethora, and sometimes conflicting, data regarding p53 pathway-mediated signaling responses also prompted quantitative, systemic analysis by mathematical modeling and bioinformatics analysis to decipher the differential control of p53 pathway activity at the collective, network level [6–9]. However, most of the computational work employs the network topology-based approach and falls short of providing mechanistic insight, in particular for understanding the divergent p53 pathway responses to different stress stimuli and in variable mammalian cell types.

Single-cell studies both by us and others showed that alteration of the induction dynamics of p53 not only is an important mechanism to modulate p53 activity but also provides a quantitative, dynamic readout to investigate the unique network motifs and collective regulatory activities of the p53 pathway, alternative to the static network topology-based approach. The control of p53 dynamics over cell fate was first examined for cellular response to transient gamma or UV irradiation and revealed intriguing periodic oscillation of p53 level [10–13]. We later uncovered other p53 dynamic modes in response to a DNA damaging drug, Etoposide, i.e., monotonic p53 induction and an extended large p53 pulse, which led to cell death and cell cycle arrest, respectively. We also found activation of the distinct p53 dynamic modes was context-specific, depending on not only the DNA damage level but also the mammalian cell type [14, 15]. However, the studies of p53 dynamics so far are mostly on response to DNA damage, and we know much less about how the p53 pathway and p53 dynamics are differentially activated by other types of stress stimuli.

On the mathematical modeling of p53 pathway dynamics, previous work largely focused on the periodic pulsing mode of p53, attributing it to a time-delay negative feedback loop between p53 and its main negative regulator and transcriptional target, Mdm2 (mouse double minute 2 homolog) [10, 16–18]. Dynamic features associated with the p53 pulsing mode, such as pulsing periods, amplitudes, and signaling noise, were thus the common basis and quantitative targets of most previous modeling analyses. In addition to the extensively studied p53-Mdm2 negative feedback loop, we identified an inhibitory interaction between ATM (Ataxia telangiectasia mutated) and Mdm2 resulting from ATM-mediated Mdm2 degradation, which synergized with the p53-Mdm2 negative feedback to constitute a positive feed-forward motif and switched the p53 dynamic mode from pulsing to monotonic induction upon increasing DNA damage level [15]. However, while simulation of this regulatory motif generated p53 dynamics largely similar to the single-cell imaging results at low- and high-DNA damage level (i.e., the extreme damage dose regime), the model predicted a unique transitional dynamic phenotype of p53 at the intermediate DNA damage level, with an initial p53 pulse followed by an elevated plateau of p53 level, which neither we nor other research groups have experimentally observed. This discrepancy between the model predictions and experimental findings so far led us to hypothesize that additional regulatory components beyond the well-known ATM/p53/Mdm2 regulatory motif is involved in generating the DNA damage-induced bimodal switch of p53 dynamics. We note that a few modeling analyses have examined the role of p53 feedback components, such as PTEN (phosphatase and tensin homolog) and Wip1 (wild-type p53-induced phosphatase 1), in modulating the p53 signaling dynamics [19–21]. These studies predicted a variety of biphasic p53 dynamics that were yet to be experimentally validated. It is also unclear whether and how these alternative p53 dynamics are differentially activated in response to different stress stimuli and in distinct cell types.

We thus set out in this study to identify and characterize distinct regulatory motifs that can give rise to variable signaling outputs of p53 pathway in a broad cellular context, using three anticancer chemotherapeutics that activate p53 via distinct mechanisms, including Etoposide (DNA damage inducer), Nutlin-3a (Mdm2 inhibitor), and 5-fluorouracil (nucleolar stress inducer), and five human cancer cell lines. Our single-cell imaging data revealed novel p53 dynamic modes that are stimulus- and cell type-dependent, in particular intriguing p53 transitional dynamics activated at intermediate drug concentrations. Importantly, it is these new p53 dynamic modes that vary significantly between drugs and cancer cell types, not the widely studied periodic pulsing mode of p53. Based on the experimental data, we constructed and simulated a number of minimal, core regulatory motifs of p53 pathway in a large parameter space, which allowed us to characterize unique feedback structures and uncover their quantitative roles in generating the distinct dose-response phenotypes of p53 dynamics in a context-specific manner. Our results also revealed previously unknown mediators of the chemo-drug actions that vary between cancer cell types and impact drug sensitivity. For instance, we found MCF7, the cell line used in most of the previous single-cell studies of p53 dynamic responses, is unique in its strong dependence on the negative feedbacks, thus exhibiting resistance to drug-induced p53 upregulation in response to all three anticancer drugs, despite the drugs’ different mechanisms. Our work provides important new insight into not only the context-specific p53 pathway control at the collective, network motif level but also a quantitative framework of generic network building blocks and their dynamic outputs for functionally dissecting other complex signaling networks.

## Results

### Stimulus- and cell type-dependent p53 dynamics

**Fig. 1:**
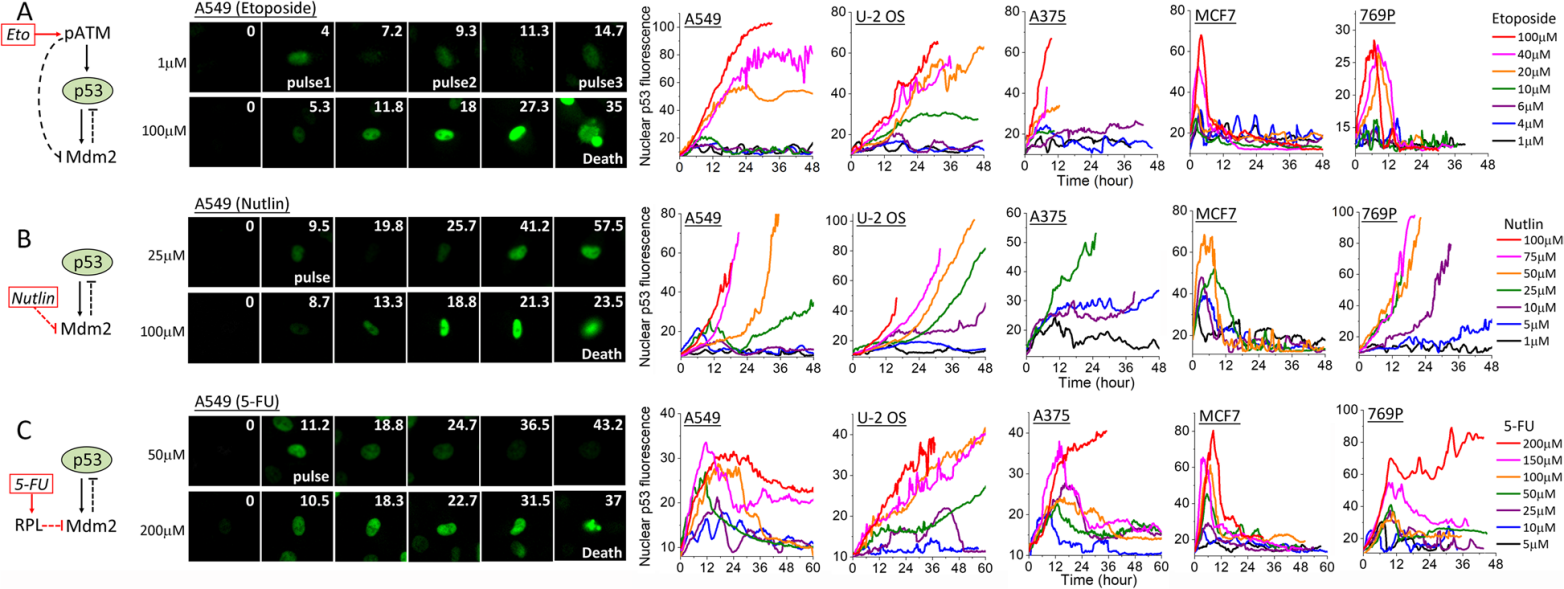
Dose responses of p53 dynamics to **A** Etoposide, **B** Nutlin-3a, and **C** 5-Fluouracil (5-FU) vary between drugs and cancer cell types. Left panel: Pathway diagrams of key regulatory components of the p53 pathway known to mediate the respective drug response. Middle panel: Still cell images of p53 fluorescence from the p53-Venue reporter in A549 cells at the indicated drug concentrations. Still images were cropped from time-lapse movies. Time (unit: hour) is indicated at the top right corner of each image. Right panel: Representative single-cell trajectories of p53 dynamics in the nucleus quantified from the p53-Venus fluorescence in the five cancer cell lines, including A549, U-2 OS, A375, MCF7, and 769-P. p53 dynamics in response to the different drug concentrations are color coded as shown on the figure. Cells were treated with the respective drug at time 0 and tracked for 48 to 60 h or till cell death occurred. The abrupt end of some p53 trajectories before 48 h, especially under high-drug concentrations, corresponds to the time of death

To experimentally profile the differential signaling outputs of p53 pathway, we used single-cell p53 dynamics acquired from live-cell imaging as the quantitative signaling readouts. We activated p53 and the p53 pathway by three different anticancer drugs. The DNA damaging drug, Etoposide, is a topoisomerase inhibitor and activates the DNA damage signaling kinase, ATM, which then phosphorylates both p53 and Mdm2, subsequently attenuating the inhibitory p53-Mdm2 interaction that enables p53 induction [22, 23] (Fig. 1A). The Mdm2 inhibitor, Nutlin-3a, directly abrogates the binding of Mdm2 to p53, thus allowing p53 level to increase [24, 25] (Fig. 1B). The anti-metabolite chemotherapeutic, 5-fluorouracile (5-FU), is a pyrimidine analog that interferes with DNA and RNA synthesis [26]. Although 5-FU induces both DNA and RNA damage, increasing evidence suggests that p53-mediated 5-FU cytotoxicity is mainly triggered by impairment of the ribosomal biosynthesis in the nucleoli [27]. Such nucleolar stress activates the translocation of ribosomal proteins, such as RPL11 and RPL5, to bind and sequester Mdm2, leading to p53 upregulation [28–30] (Fig. 1C). In addition to the different drugs, we also characterized the cell-type variation in p53 pathway response by employing five p53 reporter cancer cell lines that we generated and studied before, including A549 (lung), U-2 OS (bone), A375 (skin), MCF7 (breast), and 769-P (kidney) [15]. These cell lines were engineered to stably express a p53-Venus construct (i.e., wild-type p53 tagged with a yellow fluorescent protein, Venus) [11, 14]. Time-lapse imaging of the respective clonal fluorescent reporter lines allows us to obtain real-time p53 dynamics in individual cells via the fluorescence signal of p53-Venus, which is mostly localized in the nucleus. Representative still images and the quantified time courses of the average nuclear p53-Venus fluorescence that the majority of the cell population exhibited at each drug concentration are shown in Fig. 1 (middle and right panels) for the selected three drugs and five cancer cell lines.

The single-cell data revealed both stimulus- and cell type-dependent characteristics in the dose responses of p53 dynamics (Fig. 1 and Additional file 1: Figure S1). At low dose, Etoposide, Nutlin-3a, and 5-FU all triggered periodic pulsing of p53 in all five cell lines, although some cell lines and drugs appeared to engender more regular periodic pulsing (e.g., A549 under 1 μM Etoposide). Upon increasing drug concentrations, p53 induction changed to different dynamic modes that varied between both the drugs and cell lines. Under Etoposide, the cell population switched to either monotonic induction of p53 (A549, U-2 OS, and A375) or an extended large p53 pulse (MCF7 and 769-P), without exhibiting alternative transitional dynamics between the low- and high-drug concentrations (Fig. 1A, right panel). In contrast, responses to Nutlin-3a showed a distinctive transitional mode of p53 dynamics at the intermediate drug doses in A549, U-2 OS, and 769-P cells before a further change to the saturating, high-drug mode. Specifically, for these three cell lines, intermediate concentrations of Nutlin-3a (10–50 μM) activated an initial p53 pulse followed by approximately exponential upregulation of p53, a novel p53 dynamic mode not observed before (Fig. 1B, right panel). As Nutlin-3a concentration further increased, the initial p53 pulse diminished and p53 induction kinetics were largely exponential. Dose responses to 5-FU revealed yet another new mode of transitional dynamics in A549, A375, and 769-P, in which the initial p53 pulse was followed by an elevated plateau of p53 level that was lower than the amplitude of the initial pulse (Fig. 1C).

**Fig. 2:**
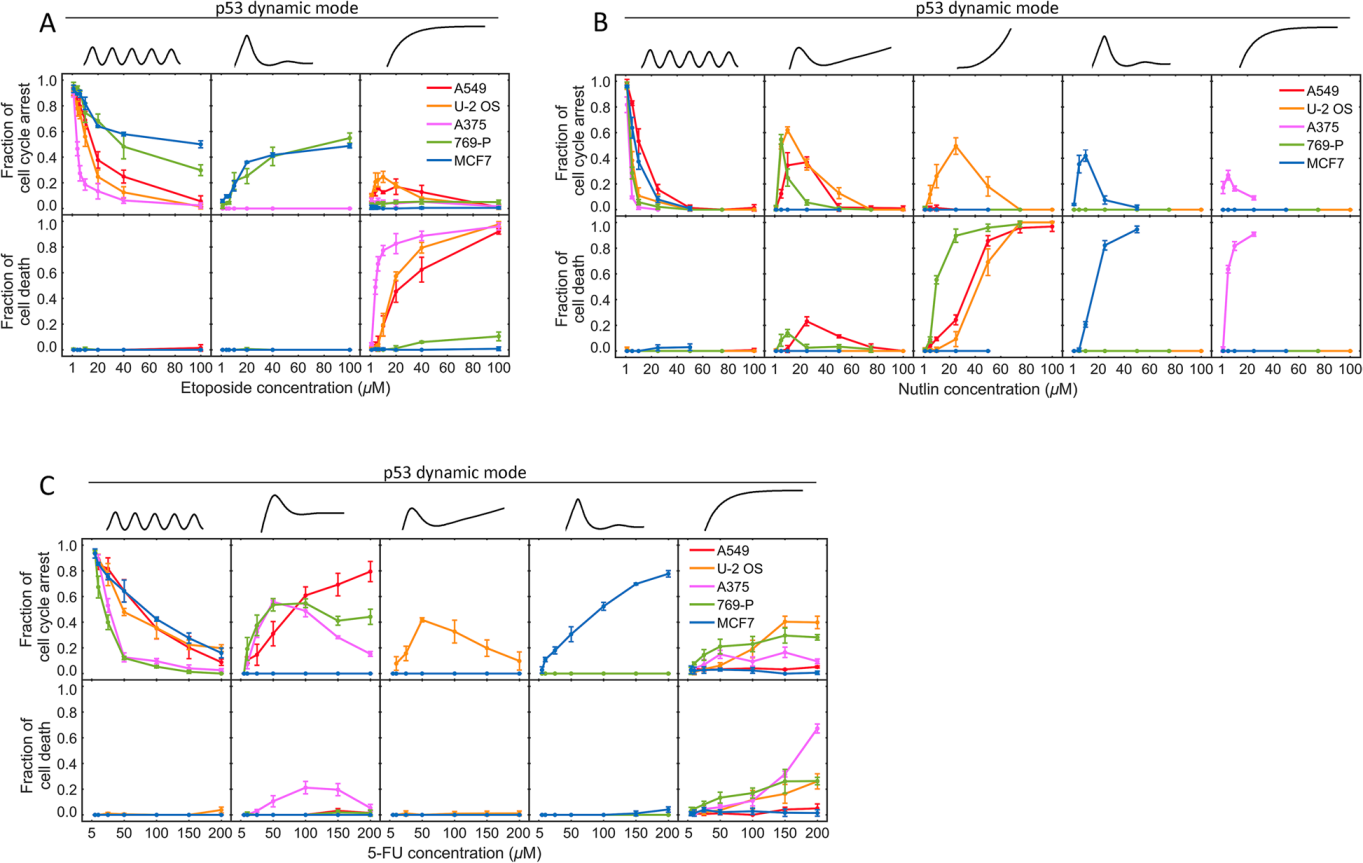
Single-cell statistics showed a correlation of distinct p53 dynamic modes with the alternative cell fate outcome of cell cycle arrest and cell death. Fractions of individual cells that exhibited the indicated p53 dynamic modes followed by cell cycle arrest (upper panels) or cell death (lower panels) were plotted as a function of the concentration of **A** Etoposide, **B** Nutlin-3a, and **C** 5-FU. The five cancer cell lines are color-coded as indicated. Data were averaged from 3 independent sets of single-cell imaging experiments. The total number of cells analyzed for each condition in each imaging experiment ranged from 48 to 156. Error bars indicate standard deviations (SDs). The individual data from each imaging experiment are provided in the Additional file 2

In addition to drug-dependent variability, significant cell-type variation in p53 responses was also observed between the five cell lines in response to the same drug that correlated with alternative cell fate outcome of cell cycle arrest and cell death. Figure 2 summarizes the fraction of cells that exhibited the different p53 dynamic modes and then went into cell cycle arrest (upper panel) or cell death (lower panel). p53 dynamics induced by Etoposide exhibited two distinct dose-response phenotypes that varied between cell types: one was from p53 pulsing at low drug concentrations to monotonic induction at high-drug concentrations (A549, U-2 OS, A375), and the other was from pulsing to an extended large pulse (MCF7 and 769-P). We have previously shown that the monotonic induction mode, which resulted in a high level of p53 upregulation, promoted drug-induced cell death, while the extended large pulse mode restrained p53 upregulation at a low level and led to mostly cell cycle arrest, rendering MCF7 and 769-P resistant to Etoposide-induced cell death even at high drug concentrations [15] (Fig. 2A). Treatment of Nutlin-3a and 5-FU resulted in five different dose-response phenotypes of p53 dynamics, including the unique transitional dynamic modes as discussed above as well as the two saturating dynamic modes as seen for Etoposide. We again observed a strong correlation between the monotonic/exponential induction mode of p53 that resulted in high p53 upregulation levels with the cell fate outcome of cell death as well as the correlation of the p53 pulsing mode with cell cycle arrest (Fig. 2B, C). Interestingly, MCF7 cells showed largely the same dose-dependent p53 dynamics in response to all three drugs, despite the different mechanisms of action of the drugs, suggesting that MCF7 may have evolved to regulate p53 upregulation by one dominant regulatory motif. Moreover, MCF7 exhibited the extended large pulse mode of p53 dynamics at saturating high concentrations of all three drugs, which led to mostly cell cycle arrest and resistance to drug-induced cell death, except for high concentrations of Nutlin-3a. In contrast, A549 and 769-P cells showed three distinctive dose responses of p53 dynamics to the three different drugs, indicating their functional dependence on a more diverse set of p53 regulatory motifs that could be potentially exploited, e.g., to confer drug selectivity. For instance, 769-P was resistant to Etoposide-induced cell death due to the suppressive, low p53 upregulation in the form of an extended large pulse. Nonetheless, p53 in 769-P can be upregulated to the high-level, monotonic induction mode by high doses of Nutlin-3a, followed by cell death (Fig. 2B), illustrating that Nutlin-3a is a more effective cytotoxic therapeutic for 769-P. Overall, the striking variability in the dose responses of p53 dynamics exhibited by the different cell lines suggested that p53 activation is regulated not only by variable drug mechanisms but also the cellular contexts of the mammalian cells, which likely have evolved differential dependence on selected p53 pathway components and interaction motifs. As the variable p53 levels resulted from the distinct p53 dynamics played a key role in promoting alternative cell fate outcomes of cell cycle arrest and cell death, as we elucidated before [15], it is important to understand the quantitative and mechanistic basis of the differential dynamic outputs of p53 pathway, which we elaborated below.

### Dynamic output of the core p53-Mdm2 negative feedback loop

To elucidate, at the network motif level, the mechanistic origin of the stimulus- and cell type-dependent dose response of p53 dynamics that we experimentally observed, we first analyzed the dynamic output of the core p53-Mdm2 negative feedback loop. Etoposide perturbs this negative feedback via phosphorylation and activation of ATM, while Nutlin-3a and 5-FU inhibit Mdm2 directly (Fig. 1, left panel). We performed computational analysis of both regulatory motifs and found their quantitative outputs of p53 dynamics were largely the same. As we already detailed the mathematical formulation of the ATM/p53/Mdm2 module in a previous study [15], here we elaborated the kinetic equations and simulation results in response to Nutlin-3a as a representative scenario. Results for the p53-Mdm2 response to Etoposide by activating the ATM/p53/Mdm2 module are provided in the supplementary materials (Additional file 1: Figure S2).

Dynamic output of the p53-Mdm2 negative feedback in response to Nutlin-3a is formulated by the following delay differential equations (DDEs).

1 $$\frac{d\left[\mathrm{p}53\right]}{dt}={k}_{\mathrm{p}0}-\frac{k_{\mathrm{mp}}}{1+{k}_{\mathrm{N}}\left[\mathrm{Nutlin}\right]}\left[\mathrm{p}53\right]\left[\mathrm{Mdm}2\right]-{\gamma}_{\mathrm{p}}\left[\mathrm{p}53\right]$$

2 $$\frac{d\left[\mathrm{Mdm}2\right]}{dt}={k}_{\mathrm{m}0}+\frac{k_{\mathrm{pm}}{\left[\mathrm{p}53\right]}_{t-{\tau}_{\mathrm{m}}}^4\ }{K_{\mathrm{pm}}^4+{\left[\mathrm{p}53\right]}_{t-{\tau}_{\mathrm{m}}}^4}-{\gamma}_{\mathrm{m}}\left[\mathrm{Mdm}2\right]$$

where [ ] denotes the dimensionless concentrations of the total proteins (p53 and Mdm2). *k*_p0_ (*k*_m0_) and *γ*_p_ (*γ*_m_) describe the rate of basal production and degradation of p53 (Mdm2), respectively. *k*_mp_ is the rate constant of Mdm2-mediated p53 degradation. The transcriptional activation of Mdm2 by the tetrameric p53 is characterized by a Hill function of 4th order with a rate constant *k*_pm_, Michaelis parameter *K*_pm_, and time delay *τ*_m_. [Nutlin] is the concentration of Nutlin-3a in the unit of μM, and *k*_N_ is the inhibitory strength of Nutlin-3a in attenuating the Mdm2-mediated p53 degradation. We set *k*_N_ = 0.45 for all of the following simulation analysis, as it produced dose responses largely similar to the experimental data.

**Fig. 3:**
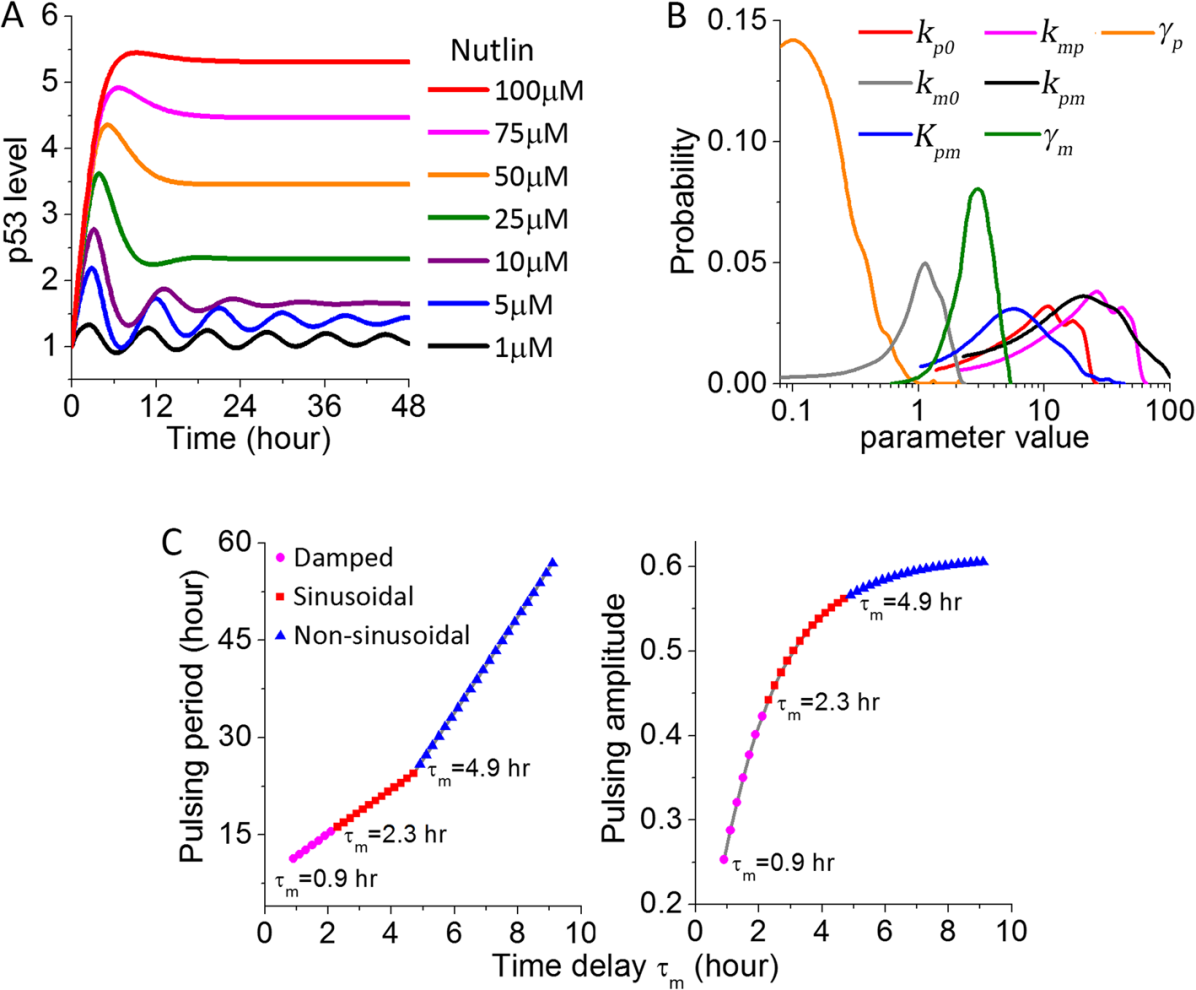
Modeling analysis of the dose-dependent output of the central p53-Mdm2 negative feedback showed a distinct mode of p53 transitional dynamics. **A** Simulation results of the dose response of p53 dynamics upon increasing Nutlin-3a concentrations. **B** Distributions of the values of the kinetic parameters involved in the p53-Mdm2 negative feedback as formulated in Eqs. (1) and (2), which can result in periodic pulsing of p53 at 1 μM Nutlin-3a. **C** Dependence of the p53 pulsing period (left panel) and pulsing amplitude (right panel) (at 1 μM Nutlin-3a) on *τ*_m_, the time delay in p53-induced Mdm2 upregulation

We first analyzed how p53 dynamics depended on the different kinetic parameters considered in Eqs. (1) and (2) by numerically simulating the DDEs in a large parameter space. This allowed us to pinpoint parameter sets that can generate periodic pulsing of p53 at 1 μM Nutlin-3a for further analysis. Under all such parameter sets, the simulated dose responses to increasing concentrations of Nutlin-3a produced similar p53 dynamics with a unique mode of transitional dynamics, i.e., an initial p53 pulse followed by an elevated plateau (Fig. 3A). Such transitional dynamics agreed with what we experimentally observed in A549, A375, and 769-P cells under the 5-FU treatment, suggesting that the p53 responses induced by 5-FU in these three cell lines were largely mediated by the central p53-Mdm2 negative feedback loop. However, this mode of p53 transitional dynamics was not observed experimentally for the dose responses to Nutlin-3a or Etoposide. Moreover, we analyzed the distribution of the parameter values that can result in pulsing p53 (Fig. 3B). The rate constants associated with p53 and Mdm2 interactions, including the p53-induced Mdm2 production rate (*k*_pm_) and the Mdm2-mediated p53 degradation rate (*k*_mp_) were most broadly distributed, spanning over a 30 to 50-fold range. This showed that the p53 pulsing output can be retained over a large variation of these two constants, indicating that the interaction structure of the p53-Mdm2 negative feedback motif largely determines the p53 pulsing output. Comparatively, the value for the Mdm2 basal degradation rates, *γ*_*m*_, exhibited the narrowest distribution, though still spanning an 8-fold range. The output of the periodic pulsing mode of p53 is thus likely most sensitive to cellular noise and/or cellular changes that alter this basal degradation rate.

Besides the above rate constants, dynamic output of the p53-Mdm2 negative feedback is also regulated by the time delay, *τ*_m_, in p53-mediated Mdm2 upregulation. In the above simulations, we set *τ*_m_ = 2.1 hr, similar to most previous modeling analysis. Varying the value of *τ*_m_ had a significant impact, in particular on the oscillatory features of p53 at low drug doses (Fig. 3C). Periodic oscillation of p53 was observed only when *τ*_m_ ≥ 0.9 h. For 0.9 h ≤ *τ*_m_ < 2.3 h, p53 dynamics displayed damped oscillation, while 2.3 h ≤ *τ*_m_ < 4.9 h gave rise to undamped, regular sinusoidal p53 oscillations. For *τ*_m_ ≥ 4.9 h, p53 oscillation became non-sinusoidal, but still maintained the periodicity. Across the whole oscillatory regime, the pulsing period of p53 was found to be linearly proportional to the time delay, *τ*_m_, and the pulsing amplitude also showed positive dependence on the *τ*_m_ value (Fig. 3C). However, varying *τ*_m_ did not alter the transitional dynamics of p53 generated by the p53-Mdm2 negative feedback as shown in Fig. 3A, again supporting that the interaction structure of the p53-Mdm2 negative feedback determines the dynamic phenotypes of p53 responses.

### p53 transitional dynamics conferred by additional positive feedback

**Fig. 4:**
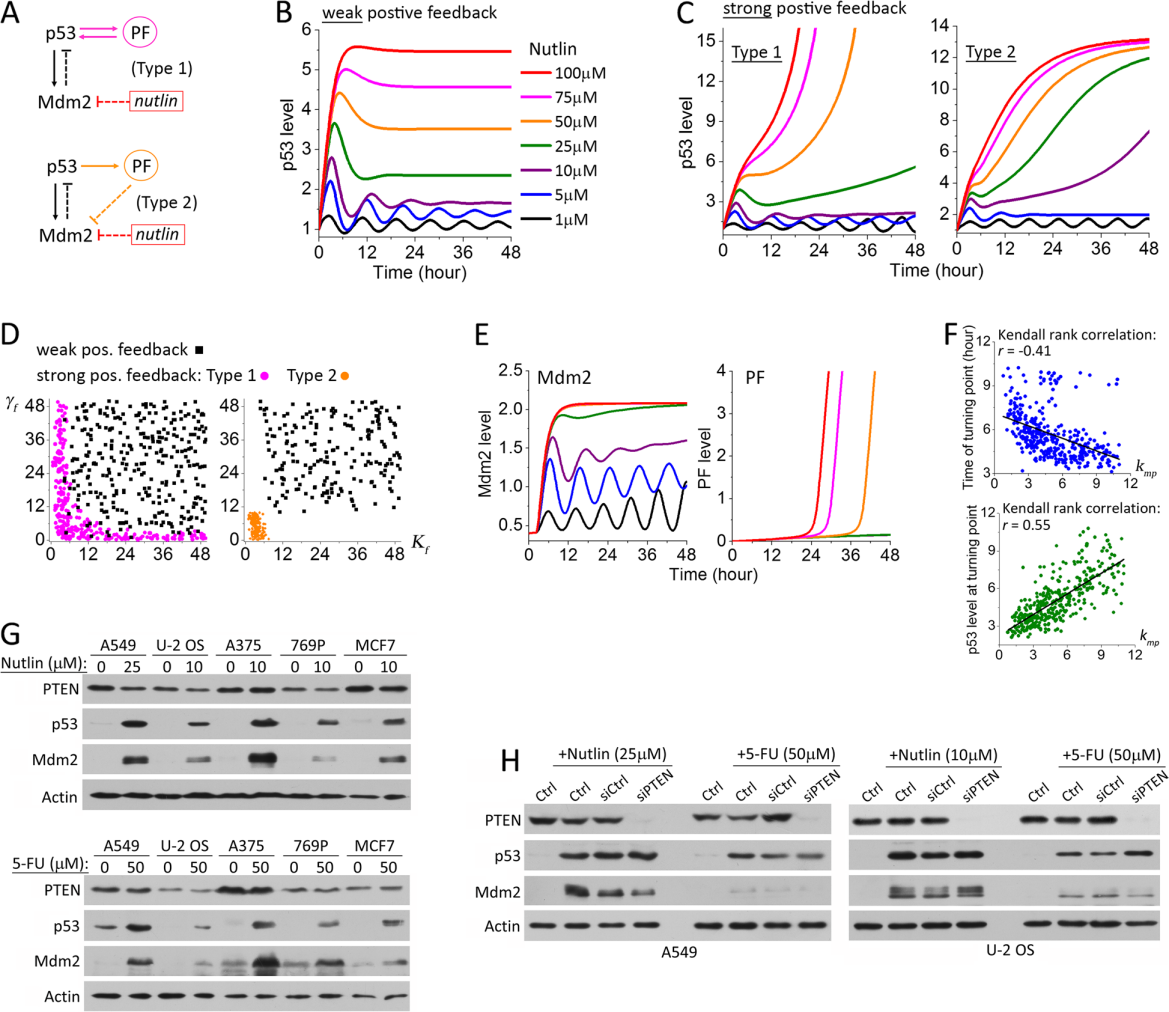
Strong positive feedback significantly alters the dose response of p53 dynamics, especially rendering unique p53 transitional dynamics. **A** Pathway diagrams with Type 1- or Type 2-positive feedback in addition to the central p53-Mdm2 negative feedback. PF denotes the positive feedback gene that is a transcriptional target of p53. **B**, **C** Dose response of p53 dynamics under **B** weak positive feedback strength and **C** strong positive feedback strength (left panel: Type 1; right panel: Type 2). **D** Correlation of the different p53 dynamic phenotypes with the values of *γ*_f_ (the degradation rate of PF) and *K*_f_ (the Michaelis parameter for p53-induced PF upregulation). **E** Dynamics of Mdm2 (left panel) and PF (right panel) resulted from the Type 1-positive feedback under increasing concentrations of Nutlin-3a. The drug concentrations are color-coded as those in **B**, **C**. **F** Correlation analysis of the time (upper panel) and p53 level (lower panel) at the turning point of p53 dynamics under intermediate concentrations of Nutlin-3a showed a significant dependence on *k*_mp_ (the rate constant of Mdm2-mediated p53 degradation). **G** Comparison of the expression levels of PTEN, p53, and Mdm2 in the five selected cancer cell lines after 24-h treatment of the indicated Nutlin-3a or 5-FU concentration. **H** p53 expression levels under PTEN knockdown (siPTEN) vs. control in A549 (left panel) and U-2 OS (right panel) cells after a 24-h treatment of the indicated Nutlin-3a or 5-FU concentration

To identify additional regulatory components and interactions needed to generate the context-dependent p53 dynamics that we experimentally observed, we first examined the impact of additional positive feedback loops. For simplicity, we chose to analyze the following two generic positive feedback structures, where p53 transcriptionally activates a downstream target gene that positively enhances p53 upregulation either directly (Type 1) or via inhibiting Mdm2 (Type 2) (Fig. 4A). Here, we showed simulation results of the dose-dependent p53 output in response to Nutlin-3a with these two different types of positive feedback loops, as p53 dynamics induced by Nutlin-3a deviated most significantly from the simulated dynamic output of the p53-Mdm2 negative feedback alone. Kinetics of the positive feedback gene, denoted as PF, and its direct activating activity on p53 or inhibitory activity on Mdm2 can be formulated as follows:

3 $$\frac{d\left[\mathrm{PF}\right]}{dt}={k}_{\mathrm{f}0}+\frac{k_{\mathrm{f}}{\left[\mathrm{p}53\right]}_{t-{\tau}_{\mathrm{f}}}^4\ }{K_{\mathrm{f}}^4+{\left[\mathrm{p}53\right]}_{t-{\tau}_{\mathrm{f}}}^4}-{\gamma}_{\mathrm{f}}\left[\mathrm{PF}\right]$$

4 $$\frac{d\left[\mathrm{p}53\right]}{dt}={k}_{\mathrm{p}0}-\frac{k_{\mathrm{mp}}}{1+{k}_{\mathrm{N}}\left[\mathrm{Nutlin}\right]}\left[\mathrm{p}53\right]\left[\mathrm{Mdm}2\right]-{\gamma}_{\mathrm{p}}\left[\mathrm{p}53\right]+{k}_{\mathrm{p}\mathrm{f}}^{\mathrm{p}}\left[\mathrm{PF}\right]\left[\mathrm{p}53\right]\ \left(\mathrm{Type}\ 1\right)$$

5 $$\frac{d\left[\mathrm{Mdm}2\right]}{dt}={k}_{\mathrm{m}0}+\frac{k_{\mathrm{p}\mathrm{m}}{\left[\mathrm{p}53\right]}_{t-{\tau}_{\mathrm{m}}}^4\ }{K_{\mathrm{p}\mathrm{m}}^4+{\left[\mathrm{p}53\right]}_{t-{\tau}_{\mathrm{m}}}^4}-{\gamma}_{\mathrm{m}}\left[\mathrm{Mdm}2\right]-{k}_{\mathrm{m}\mathrm{f}}^{\mathrm{p}}\left[\mathrm{PF}\right]\left[\mathrm{Mdm}2\right]\kern0.5em \left(\mathrm{Type}\ 2\right)$$

where *k*_f0_ and *γ*_f_ are the rate of basal production and degradation of the positive feedback gene, PF. The rate constant *k*_f_, Michaelis parameter *K*_f_, and time delay *τ*_f_ describe the transcriptional activation of PF by p53. ${k}_{\mathrm{p}\mathrm{f}}^{\mathrm{p}}$ and ${k}_{\mathrm{mf}}^{\mathrm{p}}$ are the rate constant of p53-PF interaction in the Type 1 motif and the Mdm2-PF interaction in the Type 2 motif, respectively.

For both the Type 1 and Type 2 motifs, simulations over a large parameter space returned two distinctive dose-response phenotypes of p53 dynamics. The first phenotype was common between the two motifs and largely similar to the output of the p53-Mdm2 negative feedback alone (Fig. 4B). Therefore, we attributed it to a weak positive feedback effect. The second dynamic phenotype of the p53 dose response from both the Type 1 and Type 2 motifs diverged significantly from the p53-Mdm2 core output, and we thus attributed it to a strong positive feedback effect (Fig. 4C). For the Type 1 motif, the simulated p53 dynamics under strong positive feedback were largely similar to those that we experimentally observed in A549, U-2 OS, and 769-P cells in response to Nutlin-3a. The novel p53 transitional dynamics at intermediate Nutlin-3a concentrations, i.e., an initial p53 pulse followed by an exponential increase of p53 level, were also reproduced. As for the Type 2 motif, it resulted in a different p53 transitional dynamics, in which the initial p53 pulse was followed by a sigmoidal increase of p53 level. This phenotype resembled the p53 response in U-2 OS cells under 5-FU treatment. Interestingly, we found the positive feedback strength was mainly determined by the degradation rate of PF, *γ*_f_, and the Michaelis parameter, *K*_f_ (Fig. 4D and Additional file 1: Figure S3). When either *γ*_f_ or *K*_f_ was small under the Type 1 motif, the second dose-response phenotype of p53 dynamics (denoted as the “strong pos. feedback” phenotype) was produced from the model simulation. Under the Type 2 motif, the strong positive feedback phenotype required both *γ*_f_ and *K*_f_ to be small. This result makes quantitative sense, as level of the positive feedback gene, PF, increases faster and higher when *γ*_f_ and/or *K*_f_ are small, thus resulting in stronger positive feedback effect on p53 induction.

The novel p53 transitional dynamics provide previously unexplored quantitative features to infer p53 pathway motifs and understand their functional impacts. Intuitively, the change of p53 dynamics from pulsing to an exponential or sigmoidal increase is due to the differential feedback contribution in time from the p53-Mdm2 negative feedback and the p53-PF positive feedback. In the early phase of p53 upregulation, the inhibitory effect from Mdm2 dominates, restraining p53 in a low, pulsing mode (Fig. 4E). As Mdm2 level reaches a saturating state, the upregulation of the positive feedback gene, PF, continues. At the turning point, the activating effect from PF outweighs the inhibitory effect of Mdm2, thus causing p53 dynamics to change to a different, high-level induction mode. The correlation analysis of the turning point characteristics, including the time at which the p53 dynamic mode changes and the p53 level at the point of change, with the different rate constants showed that the turning point did not depend on the rate constants associated with PF (data not shown). This further indicated that the turning point is controlled by Mdm2. Specifically, we found the turning point depended most strongly on *k*_mp_, the rate constant of Mdm2-mediated p53 degradation, pointing to the inhibitory strength of Mdm2 over p53 as the determinant of the switch of p53 dynamics in time (Fig. 4F). Overall, our simulation analysis revealed a possible dynamic origin of the new p53 transitional dynamics that we observed in response to Nutlin-3a and 5-FU and suggested that the therapeutic effects of these two drugs in some mammalian cell types are mediated by not only inhibiting Mdm2 but also activating additional positive feedback loops of the p53 pathway.

One well-known positive feedback component in the p53 pathway is PTEN, and previous modeling analyses have attributed differential p53 dynamics to PTEN activity [20, 21]. PTEN is a transcriptional target gene of p53 [31], and can functionally constitute a Type 1 positive feedback to p53 by directly binding to p53 and stabilizing it [32]. PTEN can also constitute a Type 2 positive feedback to p53 via its PIP-3 phosphatase activity that inhibits Akt kinase, leading to attenuation of the Akt-mediated Mdm2 phosphorylation and a decrease of p53 binding by Mdm2 [33]. To examine the possible involvement of PTEN as a positive feedback component to modulate p53 dynamics under the Nutlin-3a and/or 5-FU treatment, we first measured and compared the drug-induced PTEN upregulation levels in the five selected cancer cell lines. Western blot analysis showed no drug-induced PTEN upregulation in any of the cell lines (Fig. 4G). Moreover, RNAi knockdown of PTEN in A549 and U-2 OS, the two cell lines that exhibited distinctive transitional p53 dynamics, did not significantly alter the levels of p53 upregulation in response to Nutlin-3a or 5-FU (Fig. 4H) and p53 dynamics measured by single-cell imaging displayed dynamic phenotypes similar to those without PTEN knockdown as shown in Fig. 1. These results suggest that PTEN is not the key positive feedback component responsible for the differential p53 dynamics that we observed.

### p53 dynamics conferred by additional negative feedback

**Fig. 5:**
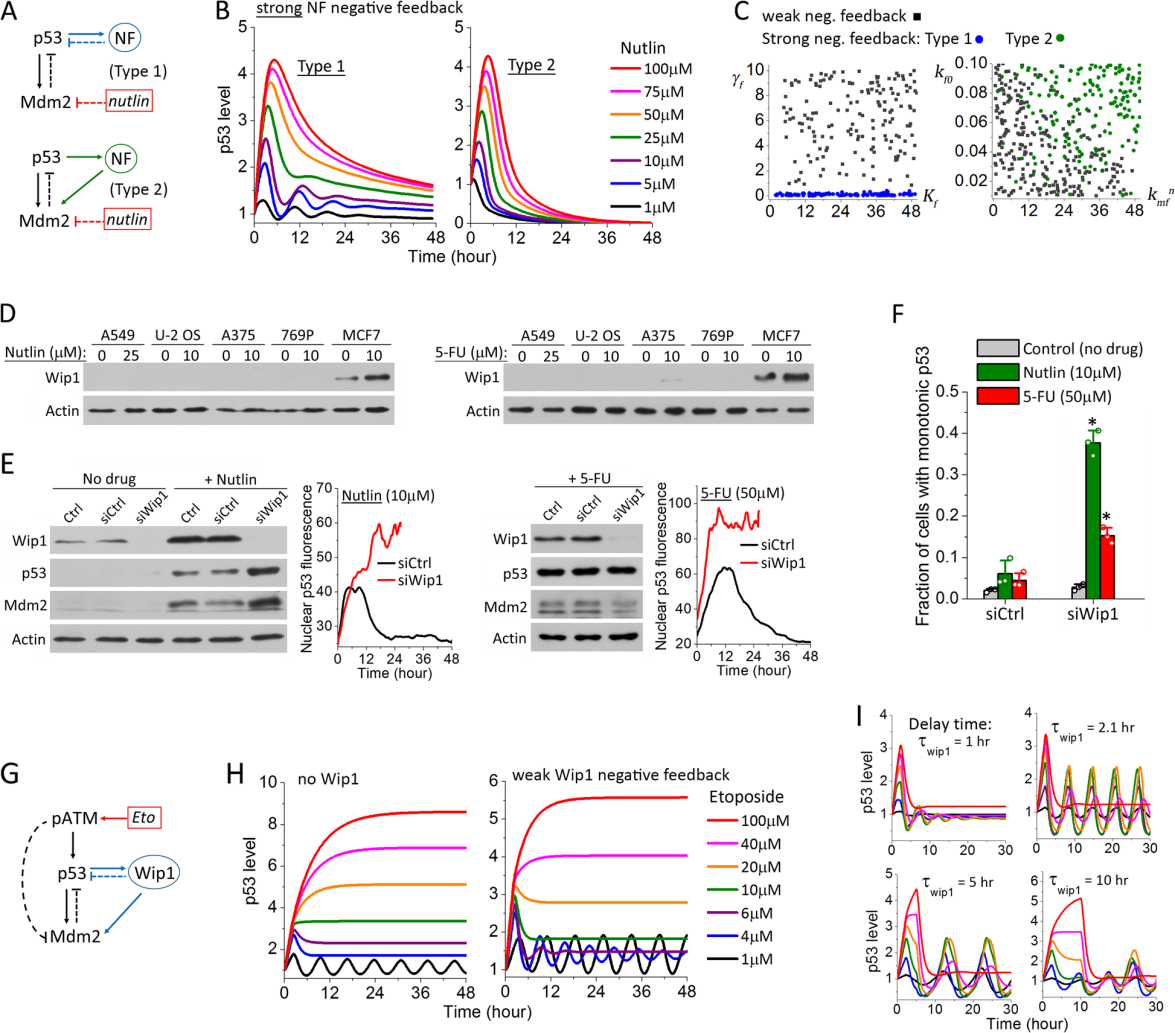
Additional negative feedback suppresses p53 upregulation by enhancing the pulsing response of p53. **A** Pathway diagrams with Type 1- or Type 2-negative feedback in addition to the central p53-Mdm2 negative feedback. NF denotes the negative feedback gene that is a transcriptional target of p53. **B** Dose response of p53 dynamics under strong negative feedback strength (left panel: Type 1; right panel: Type 2). **C** Correlation of the different p53 dynamic phenotypes with the indicated negative feedback parameters. The negative feedback strength is determined mainly by *γ*_f_ (the degradation rate of NF) for the Type 1 motif (denoted in blue), and *k*_f0_ (the basal production rate of NF) and ${k}_{\mathrm{mf}}^{\mathrm{n}}$ (the rate constant of Mdm2-NF interaction) for the Type 2 motif (green). **D** Comparison of the expression levels of Wip1 in the five selected cancer cell lines after a 24-h treatment of the indicated Nutlin-3a or 5-FU concentrations. **E** p53 expression levels and the representative single-cell trajectories of p53 dynamics under Wip1 knockdown (siWip1) vs. control in MCF7 cells after a 24-h treatment of 10 μM Nutlin-3a and 50 μM 5-FU. **F** Single-cell statistics of MCF7 cells exhibiting sustained p53 induction under the indicated RNAi and drug treatment conditions. Data were averaged from 3 independent sets of single-cell imaging experiments. The total number of cells analyzed for each condition in each imaging experiment ranged from 52 to 169. Error bars indicate standard deviations (SDs). *P* value was obtained by Student’s *t* test comparing the results under Wip1 knockdown (siWip1) with siRNA control (siCtrl). *P < 0.005. The individual data from each imaging experiment are provided in the Additional file 2 and denoted by the open circles in the figure. **G** Pathway diagram of the ATM/p53/Mdm2/Wip1 regulatory module that mediates the cellular response to Etoposide. The phosphatase, Wip1, constitutes an additional, dual negative feedback of Type 1 plus Type 2. **H** The dose response of p53 dynamics in the absence of Wip1 (left panel) and in the presence of a weak Wip1-negative feedback (right panel). **I** Detailed features of the p53 pulsing dynamics depend on *τ*_wip1_, the time delay in p53-induced Wip1 upregulation

Next, we examined the quantitative impact of additional negative feedback motif on top of the central p53-Mdm2 negative feedback loop. Again, we considered two simple scenarios, where a p53 target gene, NF, attenuates p53 upregulation either directly (Type 1) or by activating Mdm2 (Type 2) (Fig. 5A). Mathematical formulation of the additional negative feedback is largely similar to Eqs. (3)–(5), except for changing the signs of the last term in Eqs. (4) and (5) to account for the inhibitory effect of NF on p53 level. As expected, simulations of these two negative feedback motifs under weak p53-NF or Mdm2-NF feedback strength produced the same p53 dynamics as discussed above for the core module of p53-Mdm2 negative feedback alone. Under strong NF-negative feedback strength, both Type 1 and Type 2 motifs produced an extended large pulse as drug concentrations increased, with Type 2 motif resulting in sharper pulsing features (Fig. 5B). This illustrated a general effect of negative feedback in promoting p53 pulsing, retraining overall p53 induction at a low level, which we previously found to promote cell cycle arrest over cell death [15]. Different from the positive feedback motifs, we found the negative feedback strength of the Type 2 motif was determined mainly by the basal production rate of NF, *k*_f0_, and the rate constant of Mdm2-NF interaction, ${k}_{\mathrm{mf}}^{\mathrm{n}}$, and both of these rate constants had to be large in order to render a strong negative feedback effect from the Type 2 motif (Fig. 5C and Additional file 1: Figure S3).

The most widely studied negative feedback regulator that downregulates p53 level and activity is the serine/threonine phosphatase, Wip1. Wip1 is transcriptionally activated by p53 in response to various cellular stress stimuli and can directly dephosphorylate p53 at Ser15, thus forming a Type 1 negative feedback motif to attenuate p53 induction [34, 35]. Wip1 can also dephosphorylate Mdm2 at Ser395, which enhances Mdm2 affinity for p53 and the subsequent Mdm2-mediated p53 degradation, thus rendering a Type 2 negative feedback motif [36]. Of the five cancer cell lines that we profiled, MCF7 is evidently the one that strongly depends on the negative feedback regulations of p53 pathway, as it exhibits p53 pulsing similar to phenotypes shown in Fig. 5B in response to all three drugs. In our previous study, we already confirmed Wip1 played a crucial role as a negative regulator to promote p53 pulsing in MCF7 cells upon Etoposide treatment [15]. Therefore, here we only examined the involvement of Wip1 in mediating the p53 pulsing response to Nutlin-3a and 5-FU. Western blot analysis confirmed that MCF7 expressed much higher level of Wip1 than the other four cancer cell lines, and treatment of both Nutlin-3a and 5-FU induced upregulation of Wip1 in MCF7 cells (Fig. 5D). Knockdown of Wip1 by RNAi significantly enhanced p53 upregulation upon Nutlin-3a treatment (Fig. 5E, left panel), and p53 dynamics of nearly 40% MCF7 cells changed from an extend large pulse to monotonic induction (Fig. 5F). Wip1 knockdown also promoted monotonic p53 induction in response to 5-FU, though western blot analysis did not show an evident increase of the ensemble p53 induction level (Fig. 5E, right panel). This could be due to the fact that 50 μM 5-FU activated an extended large pulse of p53 in only 30% MCF7 cells, and change of p53 dynamics in about 50% of these MCF7 cells to monotonic induction by Wip1 knockdown (Fig. 5F) may not result in a large enough change in ensemble p53 level that is measurable by western blot. Overall, our results pointed to Wip1 as the key negative feedback component that promotes p53 pulsing in response to all three anticancer drugs.

In addition to the strong negative feedback phenotypes as discussed above, we found the presence of a weak negative feedback could be a possible mechanism underlying the bimodal switch of p53 dynamics that we observed for Etoposide. Simulation of the DNA damage response module (Fig. 5G), i.e., ATM/p53/Mdm2/Wip1, under weak Wip1-negative feedback strength showed a p53 dose response with a more distinct separation of the dynamic mode of p53 pulsing and monotonic induction. As shown in Fig. 5H, the central ATM/p53/Mdm2 motif without Wip1 generated p53 transitional dynamics in the form of an initial pulse followed by an elevated plateau. The presence of additional Wip1-negative feedback enhanced the pulsing characteristics at intermediate Etoposide concentrations, leading to a much lower steady-state plateau of p53 level. Given the optical and cellular noises in the experimental data, features of the p53 transitional dynamics may be further attenuated. Therefore, effects from additional weak negative feedbacks in the p53 pathway, such as via low expression of Wip1, may account for the bimodal p53 dynamics that we experimentally observed for A549, U-2 OS, and A375 in their Etoposide response, i.e., the cell population shifted from p53 pulsing directly to monotonic induction without an observable p53 transitional dynamics.

In all simulation analysis described above, we set the time delay for the additional positive and negative feedback gene to be the same as that for Mdm2, i.e., *τ*_f_ = 2.1 h. In general, we found decreasing the time delay *τ*_f_ in the positive feedback loops accelerated the switching of p53 dynamics from pulsing to exponential/sigmoidal increase, while increasing the time delay (i.e., slowing down PF upregulation) delayed and attenuated the transition. As for the negative feedbacks, shorter delay time further enhanced the suppressive effects of the negative feedback in attenuating both the p53 pulsing amplitude and the steady-state plateau level, e.g., similar to features experimentally observed in MCF7 cells (Fig. 5I, *τ*_wip1_ = 1 h). A longer delay time prolonged the pulsing period and increased the pulsing amplitude, in particular for the first pulse and at high drug concentrations, leading to a more bimodal separation of the p53 dynamic modes of periodic pulsing and an extended large pulse, which is similar to what we observed in 769-P cells under Etoposide treatment (Fig. 5I, *τ*_wip1_ = 10 h).

## Discussion

One important and reassuring finding from our modeling analysis is that the variable p53 dynamics are determined largely by the structures of the network motifs, i.e., the types of feedbacks involved and their wiring, and do not depend on the precise values of the interaction parameters. For instance, the distinctive p53 dynamic modes activated by the two types of motifs with additional positive feedback at intermediate drug concentrations are invariant over a large parameter space, as long as the feedback strength is strong. The confirmation that the motif structure determines the dynamic output of signaling pathways provides further support of the robustness of biological networks. That is, signaling response of large networks/pathways and their functional consequences can remain relatively stable, despite the presence of constant cellular noises and small environmental fluctuations that likely alter the values of multiple pathway parameters. Our results also suggest that the specific choice of parameter values in the modeling analysis is unlikely to affect the simulation results in terms of the dynamic phenotypes of p53, although the detailed quantitative features, e.g., pulsing period and amplitude, may vary. In a literature search, we found parameter values for a few key p53 pathway interactions used in different modeling papers can span two orders of magnitude. Our work provides a quantitative explanation why similar p53 dynamics can be generated with such largely different parameter values as well as a basis to compare the modeling results between these previous studies.

We showed the cell type variation in p53 dynamic response presents a new angle to identify the points of variation in different cancer cell types that confer differential drug sensitivity. Tumors are known to be highly heterogeneous both at the genotype and phenotype level. Efforts to connect genotype with phenotype so far are not successful partly because dynamic output of the signaling networks, as we showed here, can be altered by collective, moderate changes in multiple network components, rather than a large gain- or loss-of-function mutation in a single gene. We believe using the network motif approach that profiles the variable dependence of different tumor subtypes on generic network building blocks could be an alternative approach to bioinformatics analysis of large network to uncover the mechanistic origins of phenotypic heterogeneity in cancer. Moreover, dose-dependent dynamics of key signaling proteins, such as p53, can be a convenient readout to probe the cell-type-dependent motif outputs and identify the underlying points of variation.

The fact that p53 dynamics induced by Nutlin-3a cannot be recapitulated by only inhibiting Mdm2 revealed that the drug action of Nutlin-3a involves additional p53 pathway component(s) that are yet to be elucidated. We showed that in three out of the five cell lines, Nutlin-3a upregulates p53 by activating direct positive feedback (Type 1) to enhance p53 induction in addition to inhibiting Mdm2. However, it is still unclear what p53 pathway component constitutes this Type 1-positive feedback to mediate the Nutlin-3a response. We examined the most well-known positive feedback component, PTEN, which was implicated to modulate p53 dynamics by previous modeling analyses. However, our experimental results did not show dependence of p53 dynamics on either PTEN upregulation or PTEN activity under Nutlin-3a treatment. Although the molecular identity of the additional Type 1-positive feedback component involved in the Nutlin-3a response is yet to be determined in further study, the cancer cell lines that we identified to exhibit the novel p53 transitional dynamics under Nutlin-3a treatment provide the model systems to explore and understand this intriguing Type 1-positive feedback target gene of p53 [37–39].

## Conclusions

By integrating single cell dynamics data and mathematical modeling of generic network motifs, our study not only characterized novel p53 dynamic modes and the variable dose responses to distinctive anticancer chemotherapeutics but also elucidated the underlying dynamic mechanisms for differential, context-dependent p53 dynamic control at the collective regulatory motif level. We found unique p53 dynamics, in particular transitional dynamics, can be generated in a stimulus- and cell type-dependent manner by either positive or negative feedback loops that synergize with the central p53-Mdm2 negative feedback to modulate the signaling outputs of p53 pathway. Although the feedback structures that we analyzed are simple, they are already able to explain a wide spectrum of p53 dose-response dynamics that we experimentally observed at the quantitative level. Our results suggest that taking a network motif approach based on generic pathway interaction structures is an effective way to dissect and investigate the dynamic outputs of complex signaling pathways. The pathway building blocks and their unique dynamic outputs that we characterized can also be employed to gain new mechanistic insight into drug mechanism and cell type variation that impacts drug sensitivity.

## Methods

### Cell culture

Cell lines were purchased from American Type Culture Collection (ATCC, USA) and cultured under 37 °C and 5% CO_2_ in an appropriate medium supplemented with 10% of fetal calf serum (FCS), 100 U/ml penicillin, and 100 μg/ml streptomycin. A549 was maintained in F-12K, A375 in DMEM, U-2 OS in McCoy’s, and MCF7 and 769-P in RPMI. To generate fluorescent reporter cells for live-cell imaging of real-time p53 dynamics, we infected each cell line with lentiviruses encoding an established p53-Venus reporter and selected isogenic clones that exhibited drug response most similar to their respective parental line for conducting the single-cell imaging experiments. The p53-venus reporter, consisting of wild-type p53 fused to a yellow fluorescent protein, Venus, was a generous gift from Dr. Galit Lahav (Department of Systems Biology, Harvard Medical School).

### Chemicals and reagents

Etoposide, Nutlin-3a, and 5-Fluorouracil were purchased from Tocris. siRNA oligos for gene knockdown include siPTEN (GAU CAG CAU ACA CAA AUU A, used at a final concentration of 60 nM) and siWip1 (UUG GCC UUG UGC CUA CUA A, used at 30 nM). Dharmacon On-Target plus siControl (#D-001810-01) was used as non-targeting siRNA control. siRNA transfections were performed in A549 and MCF7 cells using Lipofectamine (Thermo Fisher) and in U-2 OS cells using Hiperfect (Qiagen), according to manufacturers’ instructions. Experiments were conducted after 48 h of gene silencing.

### Western blot analysis

Cell lysates were obtained using LDS sample buffer (NuPAGE, Invitrogen). Proteins were resolved on 8–15% Tris-glycine gels and transferred onto the PVDF membranes. Blots were probed with commercially available primary antibodies and chemiluminescent detection using ECL-Prime (Amersham). Antibodies used in the western blot analysis include p53 (#sc-126), Mdm2 (#sc-965), and Wip1 (#sc-376257) from Santa Cruz and PTEN (#9552) from Cell Signaling. Anti-actin (#A5316) antibody from Sigma was used as a loading control.

### Time-lapse microscopy

Cells were plated in 96-well imaging plate (μ-plate, ibidi, Germany) and cultured in phenol red-free CO_2_-independent medium (Invitrogen) supplemented with 10% FCS, 100 U/ml penicillin, and 100 μg/ml streptomycin. Cell images were acquired with the Nikon Eclipse Ti2-inverted microscope enclosed in a humidified chamber maintained at 37 °C. Cells were imaged every 10 min using a motorized stage and a 20X objective (NA = 0.75).

The real-time dynamics of nuclear p53 was analyzed based on the p53-Venus fluorescence in the nucleus. For quantifying the single-cell p53 traces, we used an automatic cell tracking program that we developed using Matlab. The program consists of image analysis procedures that sequentially segment the individual cells, track them in time, identify the nucleus, and measure the p53 fluorescence intensity in the nucleus.

### Mathematical models and computational analysis

Detailed description of the mathematical models, computational simulations, and parameter sets of the models can be found in the result section and the supplementary text in the Additional file 1.

## Supplementary Information

**Additional file 1: **Supplementary text that describes the mathematical model, simulation procedures and parameters; the supplementary Tables S1-S4. **Table S1.** Model parameters for simulating the p53 dynamic responses to Nutlin-3a. **Table S2.** Model parameters for the additional positive feedback motifs associated with PF. **Table S3.** Model parameters for the additional negative feedback motifs associated with NF. **Table S4.** Model parameters for simulating the p53 dynamic output of the ATM/p53/Mdm2/Wip1 regulatory module in response to Etoposide; and the supplementary Figures S1-S4. **Figure S1.** Additional single cell trajectories of p53. **Figure S2.** Simulation results for the ATM/p53/Mdm2 module in response to Etoposide. **Figure S3.** Correlation of the feedback parameter values with the p53 dynamic phenotypes. **Figure S4.** Full western blots.

**Additional file 2.** Excel file that provides the individual data values of the single cell statistics plotted in Fig. 2 and Fig. 5F.

## Data Availability

All data generated or analyzed during this study are included in this published article and its supplementary information files. The individual data of single-cell statistics analyzed from three replicates of live-cell imaging experiments and plotted in Figs. 2 and 5F are provided in the Additional file 2. The p53 reporter cell lines analyzed in this study were generated previously (15) and will be provided upon request.

## Abbreviations

ATM: Ataxia telangiectasia mutated
Mdm2: Mouse double minute 2 homolog
PTEN: Phosphatase and tensin homolog
Wip1: Wild-type p53-induced phosphatase 1
