# Supplementary material for: Single-cell analysis of p53 transitional dynamics unravels stimulus- and cell type-dependent signaling output motifs
Source: BMC Biol. 2022 Apr 11;20:85. doi: 10.1186/s12915-022-01290-7 (PMC9004066; doi:10.1186/s12915-022-01290-7)
Supplement: Supplementary file 1 — Additional file 1: Supplementary text that describes the mathematical model, simulation procedures and parameters; the supplementary Tables S1-S4. Table S1. Model parameters for simulating the p53 dynamic responses to Nutlin-3a. Table S2. Model parameters for the additional positive feedback motifs associated with PF. Table S3. Model parameters for the additional negative feedback motifs associated with NF. Table S4. Model parameters for simulating the p53 dynamic output of the ATM/p53/Mdm2/Wip1 regulatory module in response to Etoposide; and the supplementary Figures S1-S4. Figure S1. Additional single cell trajectories of p53. Figure S2. Simulation results for the ATM/p53/Mdm2 module in response to Etoposide. Figure S3. Correlation of the feedback parameter values with the p53 dynamic phenotypes. Figure S4. Full western blots. [file 12915_2022_1290_MOESM1_ESM.pdf]

Supplementary Information for

Single-cell analysis of p53 transitional dynamics unravels stimulus- and cell type-dependent signaling output motifs

Jun Xie[1+], Lichun Zhang[1+], Bodong Liu,[1] Xiao Liang[1] and Jue Shi[1*]

1 Center for Quantitative Systems Biology, Department of Physics and Department of Biology, Hong Kong Baptist University, Hong Kong, China

* Corresponding author: Jue Shi, jshi@hkbu.edu.hk

This supplementary file includes the supplementary text that describes the mathematical models, numerical simulation procedures and parameter space search, as well as the supplementary Tables S1-S4 and the supplementary Figures S1-S4.

I. Mathematical model and analysis of the ATM/p53/Mdm2 regulatory module in response to Etoposide

Dynamic output of the p53-Mdm2 motif in response to Etoposide is formulated by the following simplified delay differential equations (DDEs).

$$\frac{d[\mathrm{ATM_p}]}{dt} = S_{\mathrm{ea}}[\mathrm{Eto}]\left([\mathrm{ATM_t}] - [\mathrm{ATM_p}]\right) - D_{\mathrm{a0}}[\mathrm{ATM_p}] \tag{1}$$

$$\frac{d[\mathrm{p53}]}{dt} = k_{\mathrm{p0}} - k_{\mathrm{mp}}\left([\mathrm{ATM_p}]\right)[\mathrm{p53}][\mathrm{Mdm2}] - \gamma_{\mathrm{p}}[\mathrm{p53}] \tag{2}$$

$$\frac{d[\mathrm{Mdm2}]}{dt} = k_{\mathrm{m0}} + \frac{k_{\mathrm{pm}}[\mathrm{p53}]^4_{t-\tau_{\mathrm{m}}}}{K^4_{\mathrm{pm}}\left([\mathrm{ATM_p}]_{t-\tau_{\mathrm{m}}}\right) + [\mathrm{p53}]^4_{t-\tau_{\mathrm{m}}}} - \gamma_{\mathrm{m}}\left([\mathrm{ATM_p}]\right)[\mathrm{Mdm2}] \tag{3}$$

where [ ] denotes dimensionless concentrations of the total proteins (p53, Mdm2, $\mathrm{ATM_t}$) or the active, phosphorylated form of ATM ($\mathrm{ATM_p}$). Briefly, ATM is phosphorylated and activated by different concentrations of Etoposide with rate constant S_{ea} and $\mathrm{ATM_p}$ is dephosphorylated with rate constant D_{a0} (Equation (1)). $\mathrm{ATM_p}$ subsequently phosphorylates p53 and Mdm2, leading to decrease in p53-Mdm2 binding and Mdm2-mediated p53 degradation, as described by the $\mathrm{ATM_p}$-dependent rate parameter k_{mp}. k_{p0} and γ_{p} describe the rate of basal production and Mdm2-independent degradation of p53, respectively (Equation (2)). The transcriptional activation of Mdm2 by tetrameric p53 is characterized by a Hill function of 4th order in Equation (3) with rate constant k_{pm}, Michaelis parameter K_{pm} and time delay τ_{m}. Our previous work showed that $\mathrm{ATM_p}$ enhanced auto-degradation of the phosphorylated form of Mdm2, therefore the Mdm2 degradation rate γ_m is also set to be $\mathrm{ATM_p}$ dependent [15].

Equations (1)-(3) were derived with the assumption of rapid equilibrium of phosphorylation and dephosphorylation, as they are much faster than transcription and degradation.

This assumption allowed us to simplify the kinetic equations for total p53 and Mdm2 without specifying their respective phosphorylated and unphosphorylated forms, as well as to obtain the ATM_p dependence of the kinetic rates as follows (refer to the supplementary information of ref. [15] for the detailed derivation).

$$k_{mp}([ATM_p]) = k_{mp0}(\frac{D_{p0}}{S_{p0} + D_{p0} + S_{ap}[ATM_p]}) \quad (4)$$

$$K_{pm}([ATM_p]) = K_{pm0}(1 + \frac{D_{p0}}{S_{p0} + S_{ap}[ATM_p]}) \quad (5)$$

$$\gamma_m([ATM_p]) = \frac{\gamma_{m0} D_{m0} + \gamma_{m1}(S_{m0} + S_{am}[ATM_p])}{D_{m0} + S_{m0} + +S_{am}[ATM_p]} \quad (6)$$

where S_{p0} (S_{m0}) and D_{p0} (D_{m0}) are the basal phosphorylation and dephosphorylation rate constants of p53 (Mdm2), S_{ap} (S_{am}) are the ATM-mediated phosphorylation rate of p53 (Mdm2), k_{mp0} is the Mdm2-mediated p53 degradation rate constant, K_{pm0} is the Michaelis constant for p53-induced upregulation of Mdm2, and γ_{m0} and γ_{m1} are the degradation rate constants of the unphosphorylated and phosohorylated Mdm2, respectively.

Similar to the modeling results for the p53-Mdm2 negative feedback motif under Nutlin-3a, p53 levels simulated for the ATM/p53/Mdm2 module produced a unique p53 transitional dynamics at intermediate Etoposide concentration, i.e., an initial large p53 pulse followed by an elevated plateau (supplementary Fig. S1A). This again did not agree with our experimental data for the Etoposide response and suggested additional regulatory components/interactions are involved beyond the ATM/p53/Mdm2 interactions. Figure S1B showed the distribution of parameter values that can generate periodic pulsing of p53 at 1 μM Etoposide as well as monotonic p53 induction at high drug dose, as we observed experimentally for the etoposide-sensitive cell

lines. The values for rate constants of Mdm2-mediated p53 degradation (k_{mp0}), p53-induced Mdm2 production (k_{pm}) and ATM-mediated Mdm2 degradation (γ_{m1}) were the most broadly distributed, while the Michaelis constant K_{pm0} spanned a smaller, but still 10-fold range.

As expected, the dynamic output of ATM/p53/Mdm2 is also regulated by the time delay in p53-mediated Mdm2 upregulation, τ_{m}. By varying τ_{m}, we found the oscillatory feature of p53 level at low drug dose is evident only when $\tau_{\mathrm{m}} \geq 0.7$ hour (hr) (Fig. S1C, left panel). More specifically, when 0.7 hr $\leq \tau_{\mathrm{m}} <$ 1.7 hr, p53 dynamics exhibit damped oscillation, while 1.7 hr $\leq \tau_{\mathrm{m}} <$ 4.3 hr gives rise to steady sinusoidal p53 oscillation. For $\tau_{\mathrm{m}} \geq$ 4.3 hr, p53 oscillation becomes non-sinusoidal, but maintains the periodicity. Across the whole oscillatory regime, period of the p53 oscillation is proportional to the time delay τ_{m}, and the oscillation amplitude also showed largely linear dependence on τ_{m}, when $\tau_{\mathrm{m}} <$ 4.3 hr (Fig. S1C, right panel). However, varying τ_{m} again did not alter the transitional dynamics of p53 induction as discussed above.

II. Numerical simulation and parameter space search

The above model and the models discussed in the main text were numerically simulated using the Matlab built-in function dde23. We set the initial concentration of the different protein components as: [p53]=1, [Mdm2]=0.4, [PF]=0 or [NF]=0 and [ATMp]=0, at time = 0. To select the proper parameter sets for the numerical simulations of the dose responses of p53 dynamics, we first randomly generated large sets (> 100,000) of parameter values between 0.01 and 100 for the core module, and then simulated p53 dynamics using the randomly generated parameter sets. Only parameter sets that can result in periodic pulsing of p53 at low drug dose as well as a stable state at [Drug] = 0 and time = 0 were considered valid and used for further parameter space analysis and numerical simulation of the dose response of p53 dynamics.

To simulate the models with either additional positive or negative feedback component, i.e, [PF] or [NF], we selected a fixed set of parameters for the core module, and then performed parameter space search only for rate constants explicitly associated with [PF] or [NF]. Again we randomly generated large sets (> 100,000) of parameter values between 0.01 and 100 for the feedback rate constants, and then simulated p53 dynamics using the randomly generated parameter sets. Only parameter sets that can result in periodic pulsing of p53 at low drug dose as well as a stable state at [Drug] = 0 and time = 0 were used for further parameter space analysis and simulation of p53 dose response under additional feedback interactions. We defined the strength of the positive/negative feedback effect by comparing the p53 dynamic phenotypes under the core module plus feedback with that under the core module alone. If addition of the feedback to the core module does not change the dose-dependent p53 dynamics to a phenotype different from that resulted from the core module alone, we define the feedback effect as weak. If addition of the feedback significantly alters the p53 dynamics to a distinctive phenotype, we define the feedback effect as strong.

Representative dose-dependent responses of p53 dynamics shown in Figures 3-5 were acquired by simulating the models with the selected parameter sets listed in the supplementary Tables S1-S4. These specific parameter sets were chosen based on the quantitative features of our single-cell imaging data as well as the western blot data published in a previous study [15]. That is, these parameter sets produced simulation results most similar to our experimental observations.

Supplementary Table S1: Model parameters for simulating the p53 responses induced by Nutlin-3a

Parameter	Value & unit	Interpretation
k_{p0}	1.3396 h^{-1}	Basal production rate of p53
k_{mp0}	3.0285 h^{-1}	Rate constant of Mdm2-induced degradation of p53
γ_p	0.1 h^{-1}	Mdm2-independent degradation rate constant of p53
k_{m0}	0.08 h^{-1}	Basal production rate of Mdm2
k_{pm}	0.9172 h^{-1}	Rate constant of p53-induced production of Mdm2
K_{pm0}	1.61	Michaelis constant for p53-induced production of Mdm2
γ_m	0.4799 h^{-1}	Degradation rate constant of Mdm2
τ_m	2.1 h	Time delay in production of Mdm2

Supplementary Table S2: Model parameters for simulating the additional positive feedback motifs associated with PF

Type 1 motif:

Parameter	Value & unit	Interpretation
$\boldsymbol{k}_{\mathbf{f0}}$	0.005 h^{-1}	Basal production rate of PF
$\boldsymbol{k}_{\mathbf{f}}$	1 h^{-1}	Rate constant of p53-induced production of PF
$\boldsymbol{K}_{\mathbf{f}}$	10.74	Michaelis constant for p53-induced production of PF
$\boldsymbol{\gamma}_{\mathbf{f}}$	0.02 h^{-1}	Degradation rate constant of PF
$\boldsymbol{k}_{\mathbf{pf}}^{\mathbf{p}}$	11.5 h^{-1}	Rate constant of p53 enhancement by PF
$\boldsymbol{\tau}_{\mathbf{f}}$	2.1 h	Time delay in production of PF

Type 2 motif:

Parameter	Value & unit	Interpretation
$\boldsymbol{k}_{\mathbf{f0}}$	0.07 h^{-1}	Basal production rate of PF
$\boldsymbol{k}_{\mathbf{f}}$	12.16 h^{-1}	Rate constant of p53-induced production of PF
$\boldsymbol{K}_{\mathbf{f}}$	2.4567	Michaelis constant for p53-induced production of PF
$\boldsymbol{\gamma}_{\mathbf{f}}$	0.2462 h^{-1}	Degradation rate constant of PF
$\boldsymbol{k}_{\mathbf{mf}}^{\mathbf{p}}$	2.1347 h^{-1}	Rate constant of Mdm2 inhibition by PF
$\boldsymbol{\tau}_{\mathbf{f}}$	2.1 h	Time delay in production of PF

Supplementary Table S3: Model parameters for simulating the additional negative feedback motifs associated with NF

Type 1 motif:

Parameter	Value & unit	Interpretation
$\boldsymbol{k}_{\mathbf{f0}}$	0.026 h^{-1}	Basal production rate of NF
$\boldsymbol{k}_{\mathbf{f}}$	0.2831 h^{-1}	Rate constant of p53-induced production of NF
$\boldsymbol{K}_{\mathbf{f}}$	39.448	Michaelis constant for p53-induced production of NF
$\boldsymbol{\gamma}_{\mathbf{f}}$	0.018 h^{-1}	Degradation rate constant of NF
$\boldsymbol{k}_{\mathbf{pf}}^{\mathbf{n}}$	3.096 h^{-1}	Rate constant of p53 inhibition by NF
$\boldsymbol{\tau}_{\mathbf{f}}$	2.1 h	Time delay in production of NF

Type 2 motif:

Parameter	Value & unit	Interpretation
$\boldsymbol{k}_{\mathbf{f0}}$	0.094 h^{-1}	Basal production rate of NF
$\boldsymbol{k}_{\mathbf{f}}$	36.14 h^{-1}	Rate constant of p53-induced production of NF
$\boldsymbol{K}_{\mathbf{f}}$	22.646	Michaelis constant for p53-induced production of NF
$\boldsymbol{\gamma}_{\mathbf{f}}$	3.171 h^{-1}	Degradation rate constant of NF
$\boldsymbol{k}_{\mathbf{mf}}^{\mathbf{n}}$	19.83 h^{-1}	Rate constant of Mdm2 enhancement by NF
$\boldsymbol{\tau}_{\mathbf{f}}$	2.1 h	Time delay in production of NF

Supplementary Table S4: Model parameters for simulating the p53 dynamic output of the ATM/p53/Mdm2/Wip1 regulatory module in response to Etoposide

Parameter	Value & unit	Interpretation
k_{p0}	1.3396 h^{-1}	Basal production rate of p53
k_{m0}	0.08 h^{-1}	Basal production rate of Mdm2
k_{pm}	0.9172 h^{-1}	Rate constant of $p53_p$-induced production of Mdm2
K_{pm0}	0.4025	Michaelis constant for $p53_p$-induced production of Mdm2
τ_m	2.1 h	Time delay in production of Mdm2
k_{mp0}	4.038 h^{-1}	Rate constant of Mdm2-induced degradation of $p53_u$
γ_p	0.1 h^{-1}	Mdm2-independent degradation rate constant of p53
γ_{m0}	0.2579 h^{-1}	Degradation rate constant of $Mdm2_u$
γ_{m1}	7.7362 h^{-1}	Degradation rate constant of $Mdm2_p$
S_{ea}	1 $(\mu M \cdot h)^{-1}$	Rate constant of etoposide-induced phosphorylation of ATM
D_{a0}	80 h^{-1}	Rate constant of dephosphorylation of ATM
S_{p0}	0.25 h^{-1}	Rate constant of ATM-independent phosphorylation of p53
S_{ap}	0.8335 h^{-1}	Rate constant of ATM-mediated phosphorylation of p53
S_{m0}	0.0153 h^{-1}	Rate constant of ATM-independent phosphorylation of Mdm2
S_{am}	0.283 h^{-1}	Rate constant of ATM-mediated phosphorylation of Mdm2
ATM_t	16	Total amount of ATM

Weak Wip1 negative feedback strength

Parameter	Value & unit	Interpretation
k_{w0}	7.267 h^{-1}	Basal production rate of Wip1
k_{pw}	1.9067 h^{-1}	Rate constant of $p53_p$-induced production of Wip1
K_{pw0}	0.4025	Michaelis constant for $p53_p$-induced production of Wip1
γ_w	13.964 h^{-1}	Degradation rate constant of Wip1
D_{p0}	0.75 h^{-1}	Rate constant of Wip1-independent dephosphorylation of p53
D_{wp}	18.093 h^{-1}	Rate constant of Wip1-mediated dephosphorylation of p53
D_{m0}	0.5 h^{-1}	Rate constant of Wip1-independent dephosphorylation of Mdm2
D_{wm}	30.525 h^{-1}	Rate constant of Wip1-mediated dephosphorylation of Mdm2
τ_w	2.1 h	Time delay in production of Mdm2

Strong Wip1 negative feedback strength

Parameter	Value & unit	Interpretation
$\boldsymbol{k}_{\mathbf{w0}}$	2.377 h^{-1}	Basal production rate of Wip1
$\boldsymbol{k}_{\mathbf{pw}}$	24.932 h^{-1}	Rate constant of $p53_p$-induced production of Wip1
$\boldsymbol{K}_{\mathbf{pw0}}$	0.4025	Michaelis constant for $p53_p$-induced production of Wip1
$\boldsymbol{\gamma}_{\mathbf{w}}$	5.964 h^{-1}	Degradation rate constant of Wip1
$\boldsymbol{D}_{\mathbf{p0}}$	0.75 h^{-1}	Rate constant of Wip1-independent dephosphorylation of p53
$\boldsymbol{D}_{\mathbf{wp}}$	7.7107 h^{-1}	Rate constant of Wip1-mediated dephosphorylation of p53
$\boldsymbol{D}_{\mathbf{m0}}$	0.5 h^{-1}	Rate constant of Wip1-independent dephosphorylation of Mdm2
$\boldsymbol{D}_{\mathbf{wm}}$	33.118 h^{-1}	Rate constant of Wip1-mediated dephosphorylation of Mdm2

III. Supplementary figures

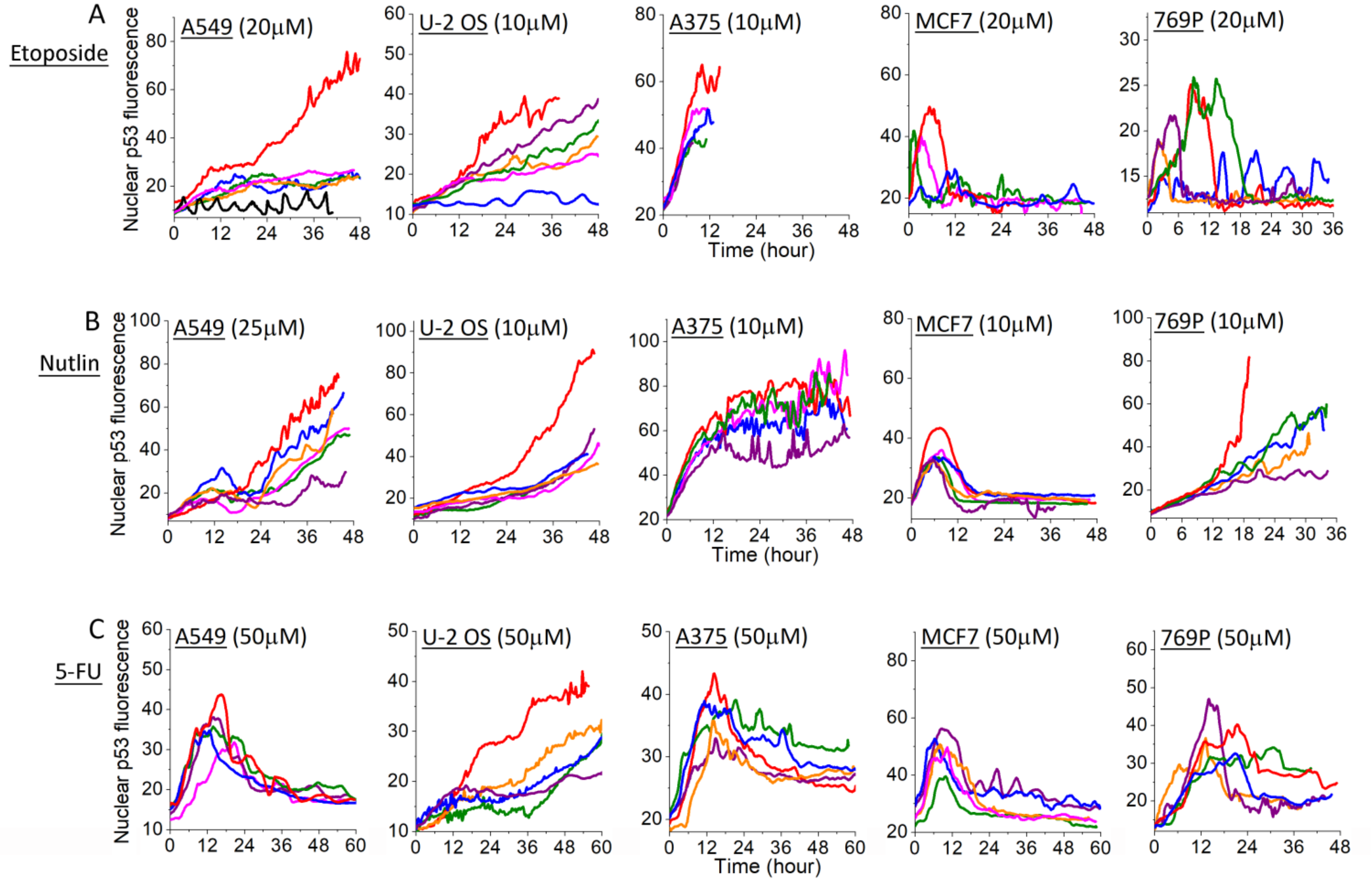

Supplementary Figure S1. Additional representative single-cell trajectories of p53 transitional dynamics in response to intermediate concentrations of (A) Etoposide, (B) Nutlin-3a, and (C) 5-FU. Cells were treated with the indicated intermediate drug concentration at time 0 and the fluorescence signal of p53-Venus was tracked for 36 to 60 hours or till cell death occurred.

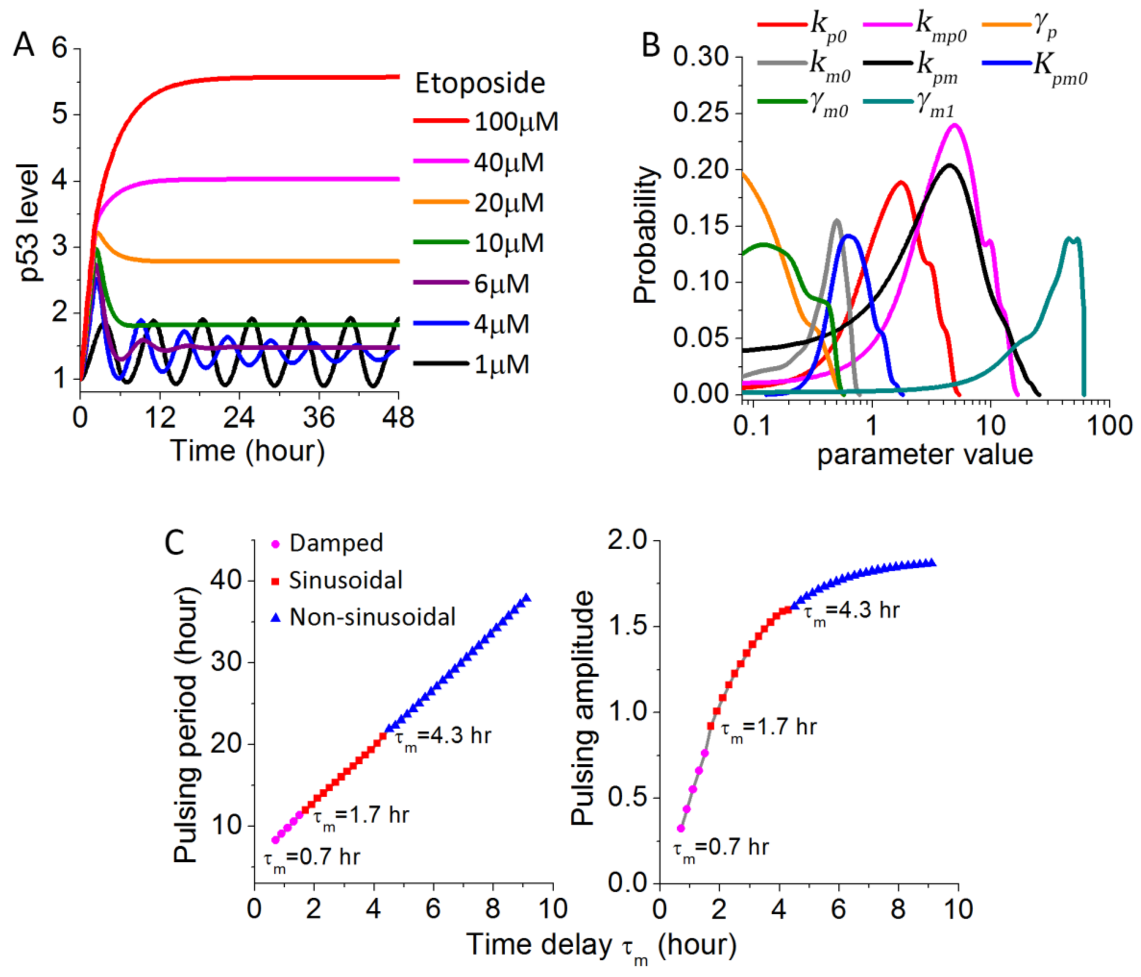

Supplementary Figure S2. Simulation results for the ATM/p53/Mdm2 module in response to Etoposide. (A) Simulation results of the dose response of p53 dynamics upon increasing Etoposide concentrations. (B) Distributions of the values of the kinetic parameters involved in the ATM/p53/Mdm2 regulatory module as formulated in Equations (1)-(3) in the supplementary text, which can result in periodic pulsing of p53 at 1 μM Etoposide. (C) Dependence of the p53 pulsing period (left panel) and pulsing amplitude (right panel) on the time delay in p53-mediated Mdm2 upregulation, τ_{m}, at 1 μM Etoposide.

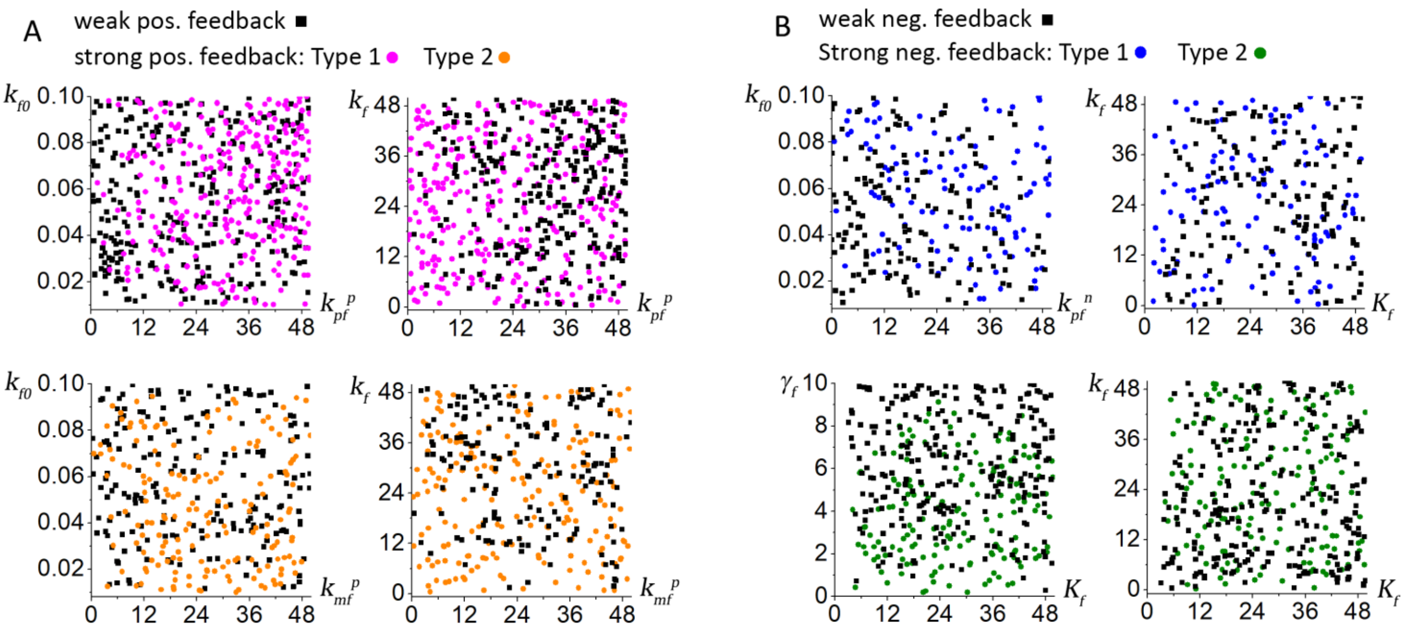

Supplementary Figure S3. Correlation analysis of the different p53 dynamic phenotypes in the presence of additional (A) positive feedback or (B) negative feedback with the indicated feedback parameters. The lack of correlation patterns in both (A) and (B) illustrated that for Type 1 motif, the positive/negative feedback strength did not strongly depend on k_{f0} (the basal production rate of PF/NF), k_{f} (rate constant of p53-induced production of PF/NF), or $k_{\mathrm{pf}}^{\mathrm{p}}$ /k_{pf}^{n} (rate constant of p53-PF/NF interaction), and the negative feedback strength also did not depend on K_{f} (the Michaelis parameter for p53-induced NF upregulation). For the Type 2 motif, the positive feedback strength did not strongly depend on k_{f0}, k_{f}, or $k_{\mathrm{mf}}^{\mathrm{p}}$ (rate constant of Mdm2-PF interaction), while the negative feedback strength did not strongly depend on k_{f}, K_{f} or γ_{f} (the degradation rate of NF).

kDa 5 cell lines_Nutlin (Fig. 4G & 5D)

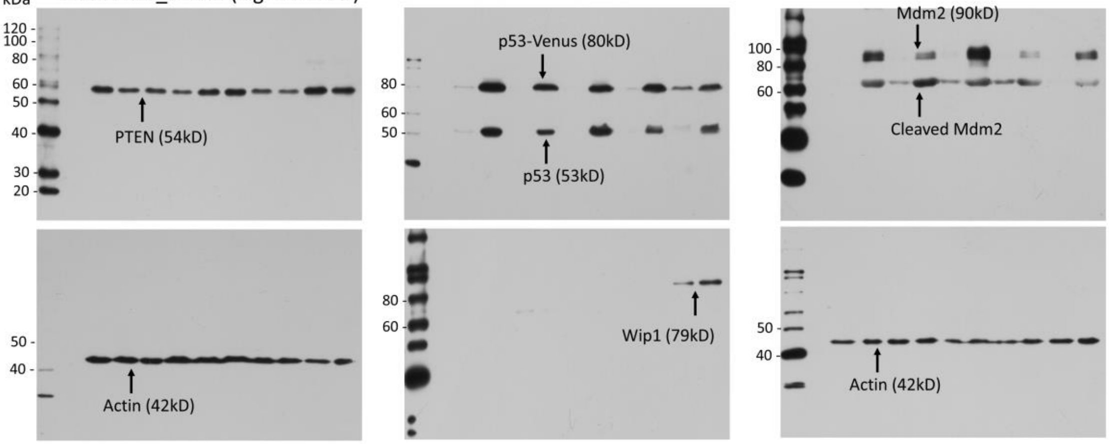

5 cell lines_5-FU (Fig. 4G & 5D)

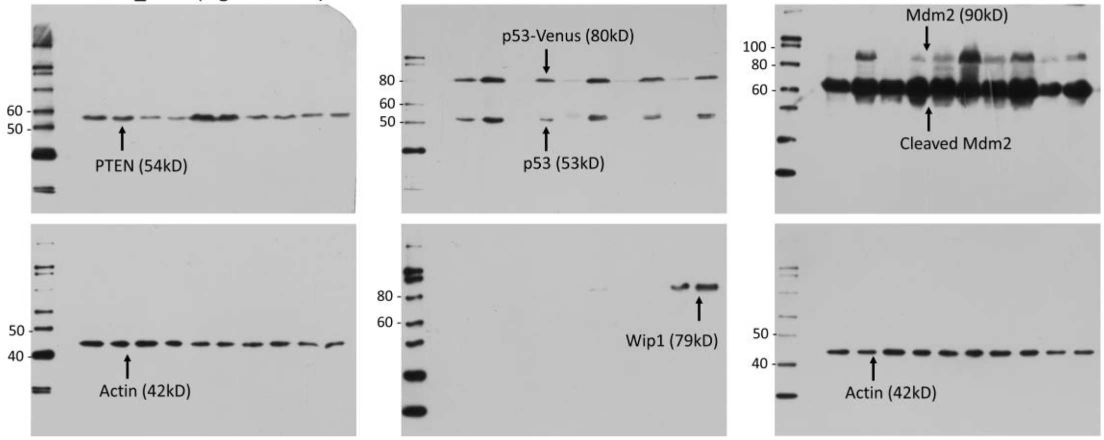

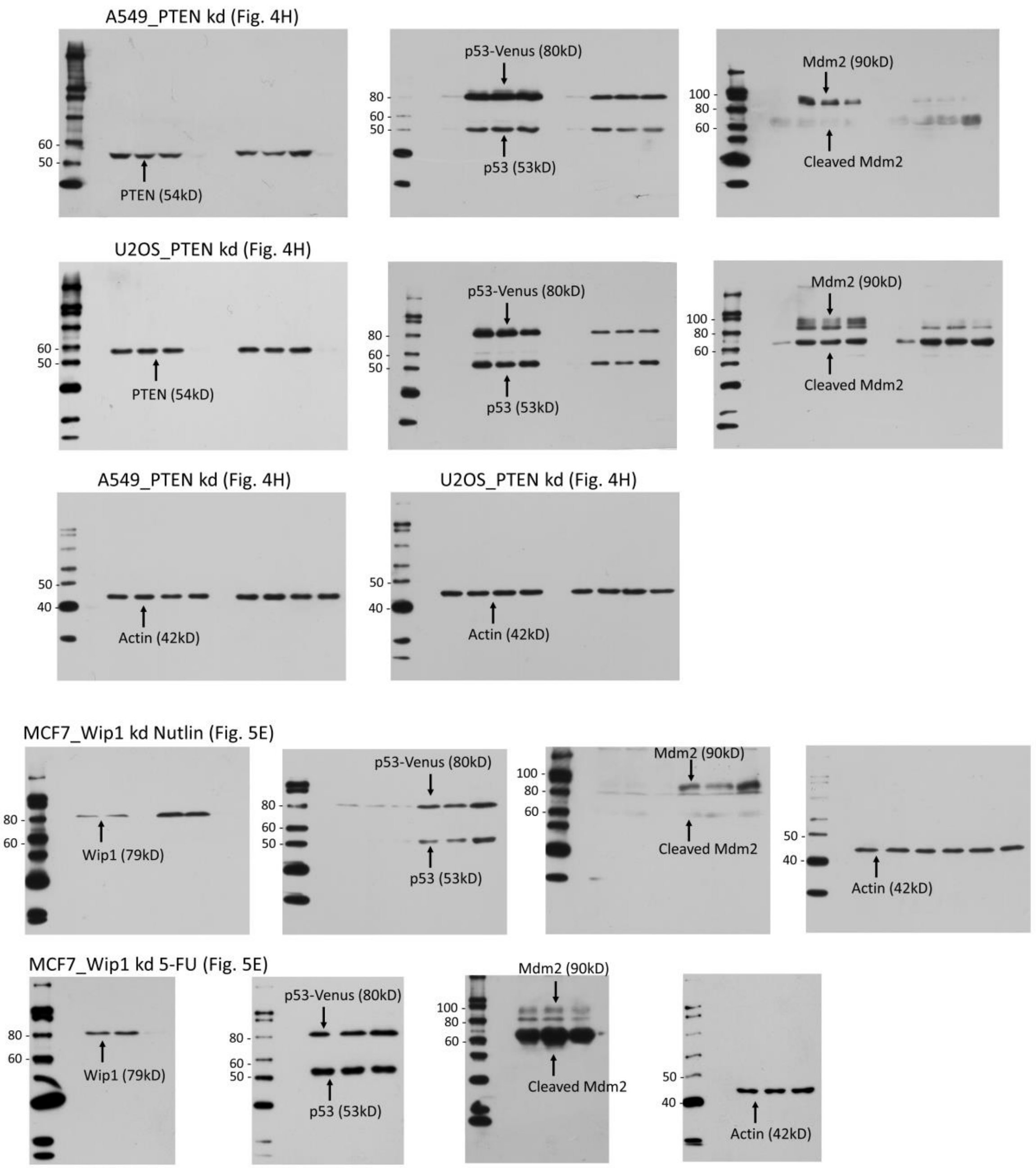

Supplementary Figure S4. Full western blots of the individual proteins shown in Figures 4G, 4H, 5D and 5E. The molecular weights are indicated by the marker (MagicMark XP Western Protein Standard, Thermo Fisher) on the left of the western blots. *The molecular weight of Wip1 is 64 kD. It runs at the nominal molecular weight of 79 kD on the western blots, the same as what the datasheet of Santa Cruz shows for this Wip1 antibody (#sc-376257).
